# Differential diagnoses of COVID-19 pneumonia: the current challenge for the radiologist—a pictorial essay

**DOI:** 10.1186/s13244-021-00967-x

**Published:** 2021-03-11

**Authors:** Alessia Guarnera, Pierfrancesco Podda, Elena Santini, Pasquale Paolantonio, Andrea Laghi

**Affiliations:** 1 Department of Surgical and Medical Sciences and Translational Medicine, Sapienza University of Rome – Sant’Andrea University Hospital, Via di Grottarossa, 1035-1039, 00189 Rome, Italy; 2 Department of Radiology, San Giovanni Addolorata Hospital, Via Dell’Amba Aradam 9, 00184 Rome, Italy

**Keywords:** COVID-19, Coronavirus, Pneumonia, Differential diagnosis, Computed tomography

## Abstract

**Background:**

COVID-19 pneumonia represents the most severe pandemic of the twenty-first century and has crucial clinical, social and economical implications. The scientific community has focused attention and resources on clinical and radiological features of COVID-19 pneumonia. Few papers analysing the vast spectrum of differential diagnoses have been published.

**Main body:**

Complexity of differential diagnosis lays in the evidence of similar radiological findings as ground-glass opacities, crazy paving pattern and consolidations in COVID-19 pneumonia and a multitude of other lung diseases. Differential diagnosis is and will be extremely important during and after the pandemic peak, when there are fewer COVID-19 pneumonia cases. The aim of our pictorial essay is to schematically present COVID-19 pneumonia most frequent differential diagnoses to help the radiologist face the current COVID-19 pneumonia challenge.

**Conclusions:**

Clinical data, laboratory tests and imaging are pillars of a trident, which allows to reach a correct diagnosis in order to grant an excellent allocation of human and economical resources. The radiologist has a pivotal role in the early diagnosis of COVID-19 pneumonia because he may raise suspicion of the pathology and help to avoid COVID-19 virus spread.

## Key points

1. COVID-19 pneumonia generally passes through four stages characterised by different radiological features.
2. Ground-glass opacities, crazy paving, consolidations and fibrotic striae are common to various pathologies.
3. Radiological differential diagnosis roots in the localisation of these radiological features in the lung and in their timing of occurrence.
4. Presence of pulmonary nodules, pleural effusions and adenopathies is suggestive of alternative diagnoses.

## Background

Since December 2019, when a novel coronavirus has been identified in Wuhan [1] and then termed COVID-19 [2], the viral pneumonia has been spreading around the globe, and on March 11, 2020, the WHO defined COVID-19 pneumonia as pandemic [3].

Since COVID-19 outbreak, the scientific community has focused on clinical and radiological features of COVID-19 pneumonia, while few papers have analysed the vast spectrum of COVID-19 pneumonia differential diagnoses [4–6].

Complexity of differential diagnosis is high, especially in populations not drastically affected by COVID-19, and will be higher after the peak of pandemic, when there are few cases.

The crucial and challenging role of the radiologist is and will be to raise suspicion of COVID-19 pneumonia, to guarantee a correct allocation of human and economic resources, and to prevent COVID-19 virus spread.

The aim of our pictorial essay is to schematically present COVID-19 pneumonia most frequent differential diagnoses to help the radiologist face the current COVID-19 pneumonia challenge.

## Main text

### COVID-19 pneumonia

COVID-19 pneumonia is a virulent pulmonary pathology, with high person-to-person transmission [7] through the inhalation of virus which infects alveolar and endothelium cells by linking to the receptor for ACE II [8].

Chest radiography has been defined as insensitive in mild or early COVID-19 infection [9, 10]; therefore, it does not represent an efficient radiological tool to reach an early and correct diagnosis. Its main role, especially as portable chest radiography and in relation to COVID-19 pneumonia, is pneumonia monitoring after diagnosis or alternative diagnosis assessment [9, 11], because it is time-effective and cost-effective and the equipment decontamination is easier and quicker compared to CT scan. These characteristics make chest radiography a useful technique to minimise the risk of cross-infection and to avoid radiological service availability disruption, which happens during CT scan decontamination [9].

CT is more sensitive for early COVID-19 pneumonia detection, disease progression monitoring and alternative diagnosis assessment [9]. In the setting of COVID-19 pandemic, the greater sensitivity of CT is pivotal to guarantee an early diagnosis and isolation of infected patients [9].

**Fig. 1:**
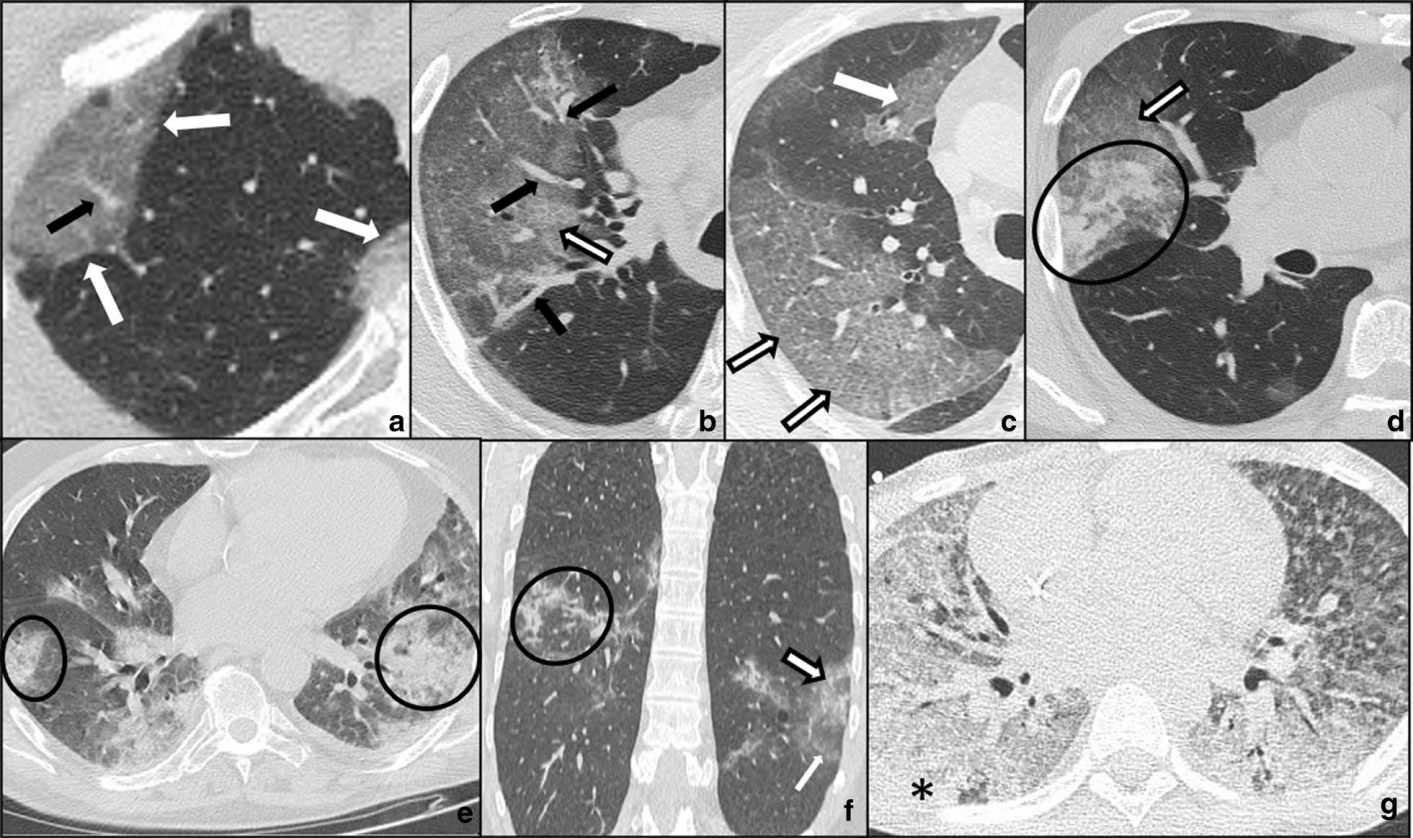
**a**–**g** COVID-19 pneumonia phases. HRTCs of patients showing the different phases and possible evolutions of COVID-19 pneumonia. In a, two subpleural, sharply demarked ground-glass opacities (white arrows in **a**) indicate phase 1. There is evidence of concomitant widening of peripheral vessels (black arrow in **a**). In **b** and **c**, subpleural extensive areas of ground glass (white arrow in **c**) and crazy paving pattern (black bordered white arrows in **b**, **c**) are referable to phase 2. Black arrows in **b** indicate dilatated segmental and subsegmental vessels. Phase 2 may also be characterised by extensive areas of crazy paving (black bordered white arrow in **d**), in which small consolidative foci are evident (black circle in **d**); In **e**, dependent and non-dependent consolidations (black circles in **e**) are hallmarks of phase 3. Phase 4 (**f**) presents as irregular consolidative foci (black circle in **f**) coexisting with confluent ground-glass opacities (white arrow in **f**) and crazy paving pattern (black bordered white arrow in **f**), whereas ARDS, which represents a possible complication COVID-19 pneumonia, is characterised by dependent consolidations (black asterisk in **g**) and widespread ground-glass attenuation

Feng Pan identified four stages of lung involvement on chest CT, even if semiological findings may coexist in phase transitions [12] (Table 1):

- Early phase (0–4 days) or stage 1: ground-glass opacities [12] (Fig. 1a);
- Progressive phase (5–8 days) or stage 2: crazy paving pattern [12, 13], extensive ground-glass opacities and small consolidations [12] (Fig. 1b–d);
- Peak phase (9–13 days) or stage 3 [12]: consolidative foci, sometimes surrounded by an halo of ground-glass (halo sign) [12] (Fig. 1e). Atoll sign or reversed halo sign [14] has been described [13];
- Absorption phase or stage 4 (≥ 14 days) [12]: ground-glass opacities and linear consolidation that may be interpreted as a process of repair and reorganisation, partially mediated by an organisational pneumonia, which is a stereotypical response to lung injury [4] (Fig. 1f).
  Further characteristic and mostly constant findings in COVID-19 pneumonia are [12, 14, 15] (Table 1):
- subpleural, bilateral or less frequently unilateral, opacities, which are commonly located in the inferior lobes;
- possible evidence of peripheral pulmonary vessel widening;
- rarity of pleural effusions, pulmonary nodules and mediastinal lymphadenopathies.

**Table 1:** Radiological findings in COVID-19 pneumonia

Stage	Phase	Timing (days)	Predominant radiological findings	Additional findings	Spatial distribution of radiological findings
1	Early	0–4	Ground-glass opacities	Peripheral vessel widening Halo sign Atoll sign or reversed halo sign Overlapping of radiological findings in different phases Rarity of: lymphadenopathies, pleuric effusions, pulmonary nodules	Bilateral Peripheral/subpleural Centroparenchymal (atypical) Lower lobes prevalence
2	Progressive	5–8	Crazy paving pattern, ground-glass opacities and small consolidations		
3	Peak	9–13	Consolidative foci		
4	Absorption	≥ 14	Ground-glass opacities and linear consolidation		

COVID-19 pneumonia has been divided into four stages, according to Feng Pan [12], depending on the timing of typical radiological findings occurrence. Spatial distribution of radiological findings and additional findings is of primary importance to reach a correct diagnosis [12, 14, 15]

Acute respiratory distress syndrome (ARDS) represents a dire complication of severe cases of COVID-19 pneumonia [12, 16] and has been speculated to be secondary to pulmonary microvascular obstructive thrombo-inflammatory syndrome [17]. Activation of the coagulation system has been shown to be relevant in the pathogenesis of ARDS, and therefore, therapy with heparin has been suggested as treatment for severe forms of COVID-19 pneumonia [18] because it decreases mortality [19].

Radiologically ARDS presents an anteroposterior gradient and dependent consolidations coexisting with widespread ground-glass attenuation [20] (Fig. 1g).

### Differential diagnoses

There is a wide spectrum of possible differential diagnoses for COVID-19 pneumonia, and it is always necessary to consider a triptych of clinical, laboratory and radiological data to reach the correct diagnosis (Table 2).

**Table 2:** Radiological features of pathologies in differential diagnosis with COVID-19 pneumonia

Pathologies		Ground glass	Crazy paving	Consolidations
Infective pneumonia	Bacterial	R (ATYPICAL)	OD/A	C (TYPICAL)
	Viral	C	OD/A	R
	Fungal: pneumocystis jiroveci pneumonia	C	R	R
	Fungal: angioinvasive aspergillosis	(HALO)	OD/A	C
Cardiovascular	Acute pulmonary oedema	C (INTERSTITIAL)	C (INTERSTITIAL)	C (ALVEOLAR)
	Acute pulmonary embolism and infarctions	C	C	C
	Vasculitis	C (HAEMORRAGE)	C (REABSORPTION)	C (MASSIVE HAEMORRAGE)
Hypersensitivity pneumonia		C	OD/A	R
Eosinophilic pneumonia	Simple pulmonary eosinophilia	C	OD/A	C
	Acute eosinophilic pneumonia	C	C	R
	Chronic eosinophilic pneumonia	R	R	C
Aspiration pneumonia	Fluid-related ab ingestis pneumonia	C	OD/A	C
	Chronic lipoid pneumonia	C	C	C
Alveolar proteinosis		OD/A	C	R

Analysed pathologies usually share at least one radiological feature, among ground-glass areas, crazy paving opacities and consolidations, with COVID-19 pneumonia. These findings may be either common or rare presentation of pathologies or be occasionally described/absent. The timing of these features presentation frequently varies with respect to COVID-19 pneumonia characteristic phases

C, common; R, rare; OD/A, occasionally described/absent

### Infective pneumonia

Respiratory infections are the most common illnesses occurring in humans [21], the most common being community-acquired pneumonia.

#### Bacterial pneumonia

**Fig. 2:**
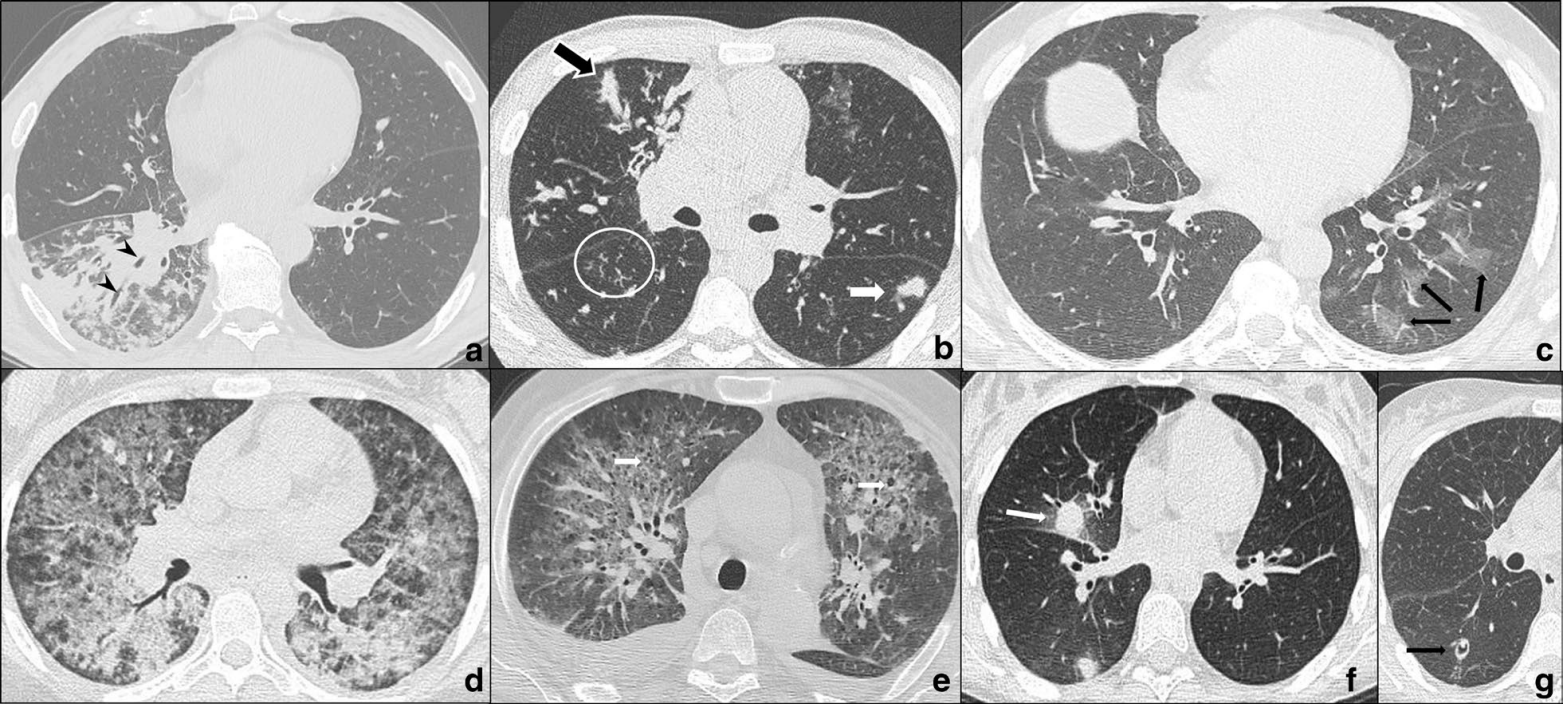
**a**–**g** COVID-19 pneumonia differential diagnosis: infective pneumonia. HRTCs of infective pneumonias. In **a**, *S. Pneumoniae* lobar pneumonia shows consolidations with air bronchogram (black arrowheads in **a**), while in **b**, atypical pneumonia presents as multiple nodular (white arrow in **b**), tubular (white bordered black arrow in **b**) consolidative foci and centrilobular nodules (white circle in **b**). In **c**, HRTC showing centroparenchymal ground-glass opacities (black arrows in **c**) in patient affected by Influenza type A pneumonia. Patients affected by HIV and pneumocistis jiroveci pneumonia in **d** and **e**, and presenting with bilateral, widespread crazy paving and ground-glass opacities on HRTC during acute pneumonia and perihilar pneumatoceles (white arrows in **e**) with pleural effusions in a long-lasting pneumonia. HRCTs of patient affected by angioinvasive aspergillosis at baseline (**f**) and after 7 days (**g**). At baseline, two consolidations rimmed by ground-glass halo are evident in **f** (white arrow in **f**, halo sign), while after therapy, excavated nodules showing “air crescent sign” (black arrow in **g**) indicate the remission phase

Radiological features for differential diagnosis:

- single consolidation with air bronchogram, usually presenting as lobar pneumonia and not exceeding pleural cleavages (typical pneumonia: *Staphylococcus aureus*, *Streptococcus pneumoniae*, *Moraxella catarrhalis*, Enterobacteriaceae) [5, 21, 22] (Fig. 2a);
- multifocal pneumonia presents with ground-glass opacities or consolidations and usually coexists with centrilobular nodules and thickening of bronchovascular bundles (atypical pneumonia: *Mycoplasma pneumoniae*, *Chlamydia pneumoniae*) [30] (Fig. 2b);
- *Mycoplasma pneumoniae* may cause multiple, bilateral ground-glass opacities as COVID-19, but mainly occurs in children [5], while COVID-19 pneumonia is less common and severe in the paediatric population [23].
- additional findings as centrilobular nodules, cavitations and pneumatoceles (more common in *S. aureus* pneumonia) [22];
- hilomediastinal lymphadenomegalies [5, 21, 22];
- pleural effusions [5, 21, 22].

#### Viral pneumonia

Viral pneumonia represents a various entity, and it is mainly the current epidemic context which suggests COVID-19 origin [4]. Treatment has been proved to be similar, currently [5].

Radiological features for differential diagnosis:

- preferential centro-parenchymal involvement (Influence type A, Adenovirus, Hantavirus) [21] (Fig. 2c);
- additional findings as centrilobular nodules and bronchial wall thickening (RSV, MERS, Influence type A) [21];
- coexisting pulmonary oedema (Hantavirus) [24];
- pleural effusions (RSV, MERS) [21];
- hilomediastinal lymphadenomegalies (Influence type A) [21].

#### Pneumocystis jiroveci pneumonia

It is an opportunistic fungal pneumonia, mostly affecting immunodeficient patients affected by AIDS or undergoing immunosuppressive therapies (Fig. 2d, e).

Anamnesis and laboratory tests are of use, but often not sufficient for a differential diagnosis with COVID-19 pneumonia. Radiological features for differential diagnosis:

- symmetrical, centroparenchymal and perihilar, confluent ground-glass opacities, generally with subpleural sparing [25] and a predilection for the upper lobes [26];
- rarity and late onset of crazy paving pattern;
- additional findings as nodules and pneumatoceles.

#### Angioinvasive aspergillosis

Angioinvasive aspergillosis is caused by Aspergillus Fumigatus [27] and generally affects immunocompromised patients with severe neutropenia [28].

Radiological features for differential diagnosis:

- ground-glass opacities and crazy paving pattern are not typical and do not precede consolidations, which frequently present a surrounding ground-glass halo (halo sign) [29] (Fig. 2f);
- in case of consolidations with no ground-glass halo and absence of other ground-glass opacities, COVID-19 pneumonia is unlikely;
- presence of air crescent sign [13, 27, 29] (Fig. 2g);
- lymphadenomegalies and pleural effusions [29].

### Cardiovascular pathologies

#### Pulmonary oedema

**Fig. 3:**
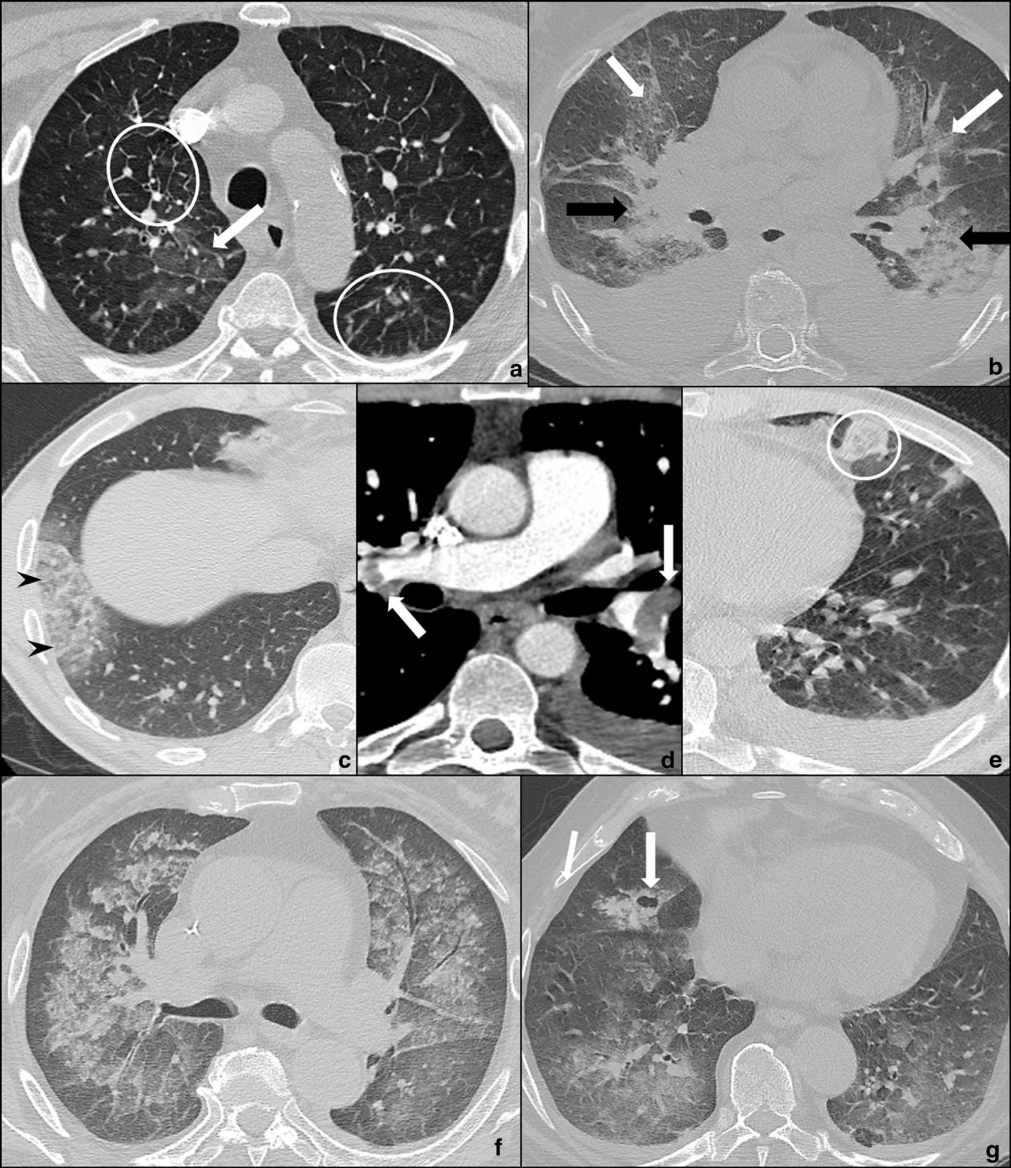
**a**–**g** COVID-19 pneumonia differential diagnosis: cardiovascular pathologies. HRCTs of patients presenting with interstitial oedema (**a**) and interstitial-alveolar oedema (**b**). In **a**, there is extensive smooth thickening of interlobular septa (white circles in **a**) coexisting with ground-glass opacities (white arrow in **a**); in **b**, confluent perihilar consolidations (black arrows in **b**) coexist with crazy paving pattern (white arrows in **b**) and bilateral pleural effusions. HRTC (**c**, **e**) and AngioCT (**d**) in patients affected by APE: bilateral emboli (white arrows in **d**) caused two pulmonary infarcts manifesting, respectively, as an area of crazy paving (black arrowheads in **c**) and a consolidation with a hypodense core (“reversed halo sign”, white circle in **e**). HRTC of a patient affected by Granulomatosis with polyangiitis (**f**, **g**) showing massive centroparenchymal DAH (**f**) and an excavated nodule (white arrow in **g**)

Two pathophysiologic and radiologic phases are recognised in cardiogenic pulmonary oedema development [30]: interstitial (Fig. 3a) and alveolar (Fig. 3b).

Apart from anamnesis, radiological features for differential diagnosis are [4, 30]:

- possible coexistence of ground-glass opacities, crazy paving pattern and consolidations with different timing of occurrence respect to COVID-19 pneumonia, crazy paving being the first pattern to be appreciated in interstitial oedema;
- diffuse, bilateral, centro-parenchymal crazy paving and ground-glass opacities with subpleural preservation. Consolidations are late findings and generally coexist with pleural effusions;
- bilateral pleural effusions, more evident in the alveolar phase of oedema;
- mediastinal lymphadenomegalies;
- cardiomegaly.

Chen et al. [31] reported acute myocarditis related to COVID-19 infection. The overlapping of findings between COVID-19 pneumonia and pulmonary oedema should always raise suspicion of myocarditis, especially in young patients [4].

#### Acute pulmonary embolism (APE)

APE, which is is commonly secondary to a phlebothrombosis, most frequently of the lower limbs, has an acute clinical onset [32] and may cause pulmonary infarctions (Fig. 3c, e).

It is crucial to collect a proper anamnesis and perform radiological examinations as AngioCT to identify luminal defects of pulmonary vessels (Fig. 3d). Radiological features for differential diagnosis are [32]:

- different phases of infarction maturation, in correlation to the onset time;
- segmental shape of infarctions are located in the vascular territories of the occluded vessels;
- presence of vessel embolus, vessel occlusion and residual peripheral clot deposition.

It has been speculated on extensive pulmonary thromboembolism in severe cases of COVID-19 pneumonia [17, 32, 33], mediated by COVID-19 endothelial tropism [8]. Luckily, heparin, which is the first therapy for APE, has shown to have effect on patients affected by severe forms of COVID-19 pneumonia [18, 19].

COVID-19 pneumonia and pulmonary embolism may coexist; in particular, a person might present with the symptoms of acute pulmonary embolism and actually suffer of fulminant pulmonary embolisation but be also infected with COVID-19 being asymptomatic from the infection [34]. GGO with a typical appearance on CT might be the only sign in this person [34].

#### Vasculitis

Vasculitis is an inflammatory process in which immune effector cells infiltrate blood vessels and surrounding tissues [35, 36]. ANCA-associated small vessel vasculitis frequently present with predominant pulmonary involvement and may cause diffuse alveolar haemorrhage (DAH) [13, 37] (Fig. 3f, g).

Anamnesis and laboratory tests may be useful to prove a clinical suspect. Radiological features for differential diagnosis are:

- DAH, presenting as ground-glass opacities or consolidative foci if bleeding is massive, is more prominent in the perihilar region and in the inferior lobes [13, 37];
- DAH presents different timing of presentation, crazy paving being the last pattern to be appreciated [37, 38];
- symmetric, bilateral ground-glass opacities or consolidations, which tend to be migratory or transient (EGPA: eosinophilic granulomatosis with polyangiitis) [13, 37];
- typical vasculitis features, as pulmonary artery aneurysms and excavated nodules may be present and are more common in granulomatosis with polyangiitis, but rarely present in EGPA [13, 37];
- coexistence of bronchial and tracheal thickening in granulomatosis with polyangiitis;
- coexistence of bronchial and bronchiolar thickening or centrilobular nodules in EGPA [37, 38];
- pulmonary oedema findings, secondary to cardiac involvement in EGPA [10, 26];
- pleural effusions [13, 37].

### Hypersensitivity pneumonia

It is an interstitial pathology caused by repetitive inhalation of and sensitization to a wide range of inorganic and organic antigens [39] in relation to occupational or environmental reasons. Usually it is divided into acute, subacute and chronic forms.

**Fig. 4:**
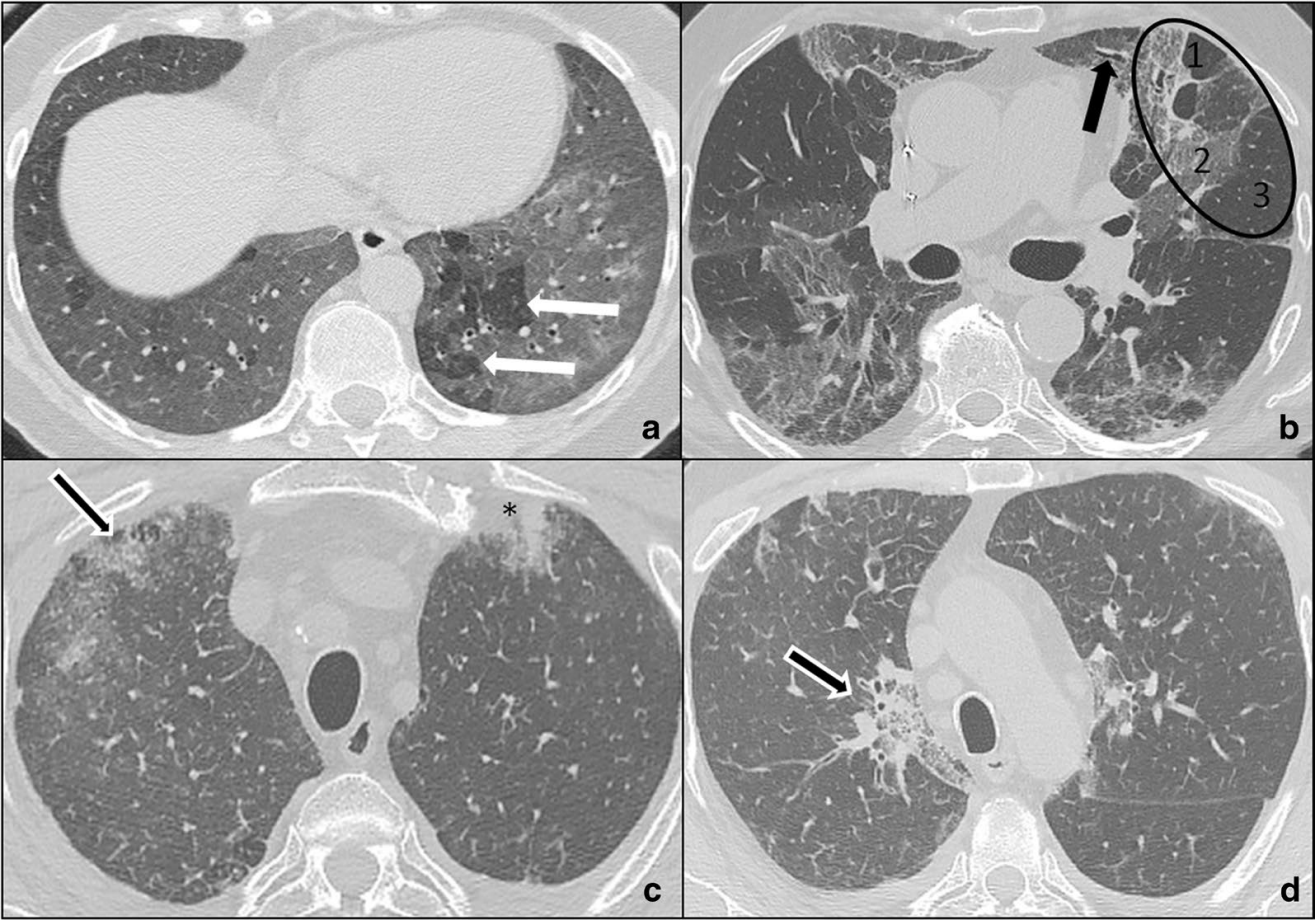
**a**–**d** COVID-19 pneumonia differential diagnoses: hypersensitivity pneumonia and eosinophilic pneumonia. HRTCs of a patient affected by hypersensitivity pneumonia at baseline (**a**) and after two years (**b**): acute/subacute hypersensitivity pneumonia is characterised by diffuse ground-glass opacities clearly demarcated from air trapping areas (white arrows in **a**); chronic hypersensitivity pneumonia presents with architectural distortions and traction bronchiectases (black arrow in **b**). The triad of mosaic oligemia (1 in **b**), ground-glass opacities (2 in **b**) and normal pulmonary parenchyma (3 in **b**) is defined as “headcheese sign” (black oval in **b**). HRTC of acute eosinophilic pneumonia (**c**, **d**) showing confluent crazy paving opacities (white bordered black arrows in **c**, **d**) coexisting with consolidations (black asterisk in **c**) and slight bilateral pleural effusions

Radiological features for differential diagnosis (Fig. 4a):

- centroparenchymal and centrilobular ground-glass opacities [40];
- rarity of crazy paving pattern and consolidations [41, 42];
- coexistence of other radiological findings as mosaic oligemia, cysts, centrilobular emphysema and centrilobular micronodules [40, 43];
- “headcheese sign,” which represents the coexistence of patchy ground-glass opacities, preserved pulmonary regions, and air trapping on HRCT [44] (Fig. 4b);
- centrilobular fibrosis, architectural distorsions, traction bronchiectases and honeycombing in the chronic stage [41, 45];
- mediastinal lymphadenomegalies.

### Eosinophilic pneumonia

Eosinophilic pneumonia represents a various group of pulmonary disorders associated with peripheral or tissue eosinophilia. Laboratory tests and anamnesis are crucial for a correct differential diagnosis [46–48]. HRTC is important to raise suspicion and avoid misdiagnoses.

Radiological features for differential diagnosis are (Fig. 4c, d):

- migrant lesions with absence of crazy paving pattern in SPE (simple pulmonary eosinophilia, also known as Loeffler syndrome) [46];
- pleural effusions, centrilobular nodules and thickening of bronchovascular bundles in AEP (acute eosinophilic pneumonia) [46, 49, 50];
- centrilobular consolidations with rarity of ground-glass opacities and crazy paving pattern in CEP (chronic eosinophilic pneumonia) [46, 51, 52];
- presence of additional findings in CEP as nodules, atelectasis, band-like opacities and pleural effusions [46, 51].

### Aspiration pneumonia

It is caused by the aspiration of different substances into the airways and lungs [53]. Radiological findings may vary, but frequently anamnesis is sufficient for the diagnosis.

To the purpose of our paper, we will focus on fluid aspiration pneumonia and on lipoid pneumonia.

#### Fluid aspiration pneumonia

**Fig. 5:**
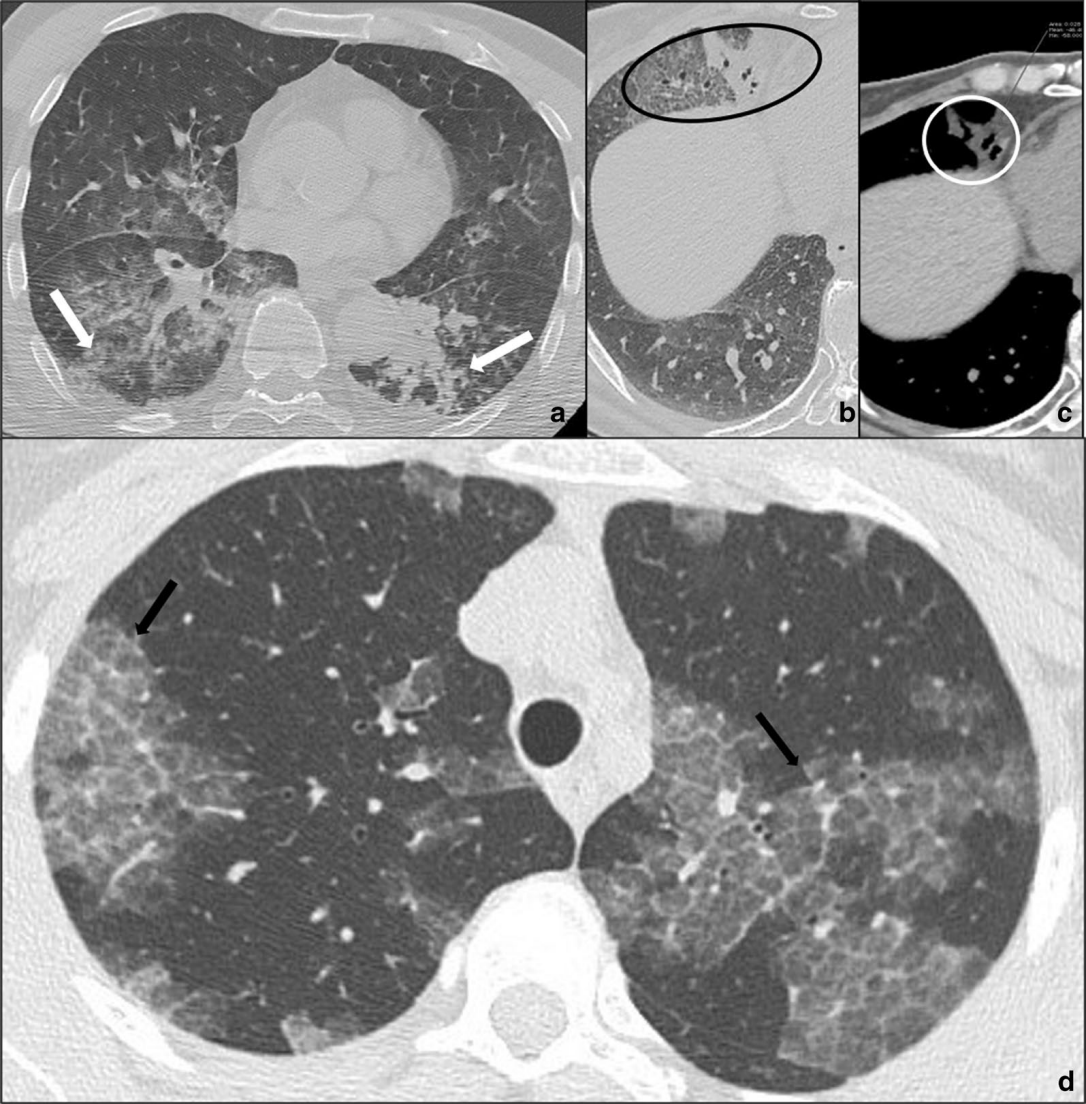
**a**–**d** COVID-19 pneumonia differential diagnoses: aspiration pneumonia, pulmonary alveolar proteinosis. HRTCs of two patients affected by fluid aspiration pneumonia presenting with bilateral, dependent and confluent consolidative foci (white arrows in **a**) and chronic lipoid pneumonia (**b**, **c**), showing a low density consolidation (mean:- 46UH in white circle in **c**) surrounded by a crazy paving halo (black oval **b**). Pulmonary alveolar proteinosis is characterised by bilateral areas of crazy paving (black arrows in **d**) and a peculiar juxtaposition of severely affected secondary lobules and normal secondary lobules

Frequently, these patients are dysfagic and their meals are liquid. Radiological features for differential diagnosis are [54] (Fig. 5a):

- dependent ground-glass opacities;
- in the late phases, with recurrence of aspiration, fibrotic architectural distortions.

#### Lipoid pneumonia

It is an acute or chronic, reactive pneumonia resulting from endogenous lipid accumulation or from exogenous lipid aspiration [55, 56]. Chronic lipoid pneumonia requires differential diagnosis with COVID-19 pneumonia, while anamnesis is generally sufficient for a clear diagnosis of acute pneumonia.

Anamnesis of lipid inhalation or fat storage disease is necessary to diagnose a lipoid pneumonia. Comparison with previous chest exams is suggested.

Radiological features for differential diagnosis are (Fig. 5b):

- ground-glass opacities and consolidations predominantly involve the middle and inferior lobes [54];
- consolidations typically present very low CT attenuation in relation to their fat content [57] (Fig. 5c);
- fibrosis during chronic stage [55, 58].

### Pulmonary alveolar proteinosis (PAP)

It is a syndrome caused by progressive accumulation of surfactant in pulmonary alveoli and may be primary in most cases or secondary to toxic inhalation syndromes, haematologic neoplasms and immune deficiency [59, 60]. Anamnesis, laboratory tests and comparison with previous HRCTs are helpful.

Radiological features for differential diagnosis are (Fig. 5d, e):

- mainly centro-parenchymal and perihilar crazy paving areas [61];
- juxtaposition of severely affected secondary lobules and normal secondary lobules;
- rarely, consolidations with air bronchogram in severe forms [61];
- fibrotic alterations in advanced stage;
- pleural effusions, cardiomegaly and lymphadenomegalies, which are features of complicated PAP.

### Drug-induced lung disease

Anamnesis of drug taking or recreative drug abuse is crucial for diagnosis and institution of appropriate treatment [62].

Among the most frequent causative drugs and the related most common pulmonary diseases we cite [62, 63]: amiodarone and methotrexate (organising pneumonia) [64]; immunosuppressants as sirolimus and everolimus (hypersensitivity pneumonia) [65]; heroin (eosinophilic pneumonia, pulmonary haemorrhage, pulmonary oedema) [66–68]; cocaine (pulmonary oedema) [69].

Radiological features for differential diagnosis of cited pathologies have already been analysed in the previous chapters.

## Conclusions

COVID-19 pneumonia early diagnosis is crucial to avoid the spread of the virus in a disease that may range from asymptomatic to extremely severe and to favour an optimal allocation of human and economic resources. In this context, the role of the radiologist is pivotal and challenging, because he filters patients and identifies possible cases of COVID-19 pneumonia.

## Data Availability

Not applicable.

## Abbreviations

AEP: Acute eosinophilic pneumonia
APE: Acute pulmonary thromboembolism
ARDS: Acute respiratory distress syndrome
CEP: Chronic eosinophilic pneumonia
DAH: Diffuse alveolar haemorrhage
EGPA: Eosinophilic granulomatosis with polyangiitis
PAP: Pulmonary alveolar proteinosis
SPE: Simple pulmonary eosinophilia
